# Gaining acceptance in next generation PBK modelling approaches for regulatory assessments – An OECD international effort

**DOI:** 10.1016/j.comtox.2021.100163

**Published:** 2021-05

**Authors:** Alicia Paini, Yu-Mei Tan, Magdalini Sachana, Andrew Worth

**Affiliations:** a European Commission, Joint Research Centre (JRC), Ispra, Italy; b U.S. Environmental Protection Agency, Office of Pesticide Programs, Research Triangle Park, NC 27709, USA; c Environment Health and Safety Division, Organisation for Economic Cooperation and Development (OECD), Paris, France

**Keywords:** PBK model, Risk assessment, OECD, Harmonisation, Human and environmental safety assessment

## Abstract

Physiologically Based Kinetic (PBK) models are valuable tools to help define safe external levels of chemicals based on internal doses at target organs in experimental animals, humans and organisms used in environmental risk assessment. As the toxicity testing paradigm shifts to alternative testing approaches, PBK model development has started to rely (mostly or entirely) on model parameters quantified using *in vitro* or *in silico* methods. Recently, the Organisation for Economic Cooperation and Development (OECD) published a guidance document (GD) describing a scientific workflow for characterising and validating PBK models developed using *in vitro* and *in silico* data. The GD provides an assessment framework for evaluating these models, with emphasis on the major uncertainties underlying model inputs and outputs. To help end-users submit or evaluate a PBK model for regulatory purposes, the GD also includes a template for documenting the model characteristics, and a checklist for evaluating the quality of a model. This commentary highlights the principles, criteria and tools laid out in the OECD PBK model GD, with the aim of facilitating the dialogue between model developers and risk assessors.

## Highlights

• Traditionally, Physiologically Based Kinetic (PBK) models have relied on *in vivo* kinetic data.
• The OECD recently published guidance for validating PBK models developed using *in vitro* and *in silico* data.
• A structured assessment framework should facilitate the dialogue between model developers and risk assessors.
• Validation strategies are provided for data-poor situations, when *in vivo* kinetic data are lacking.

## 1 Background

Physiologically based kinetic (PBK) models predict target tissue doses in defined species and under relevant exposure conditions, thus allowing for refinements in risk assessment by supporting science-based applications such as inter-species, high-to-low dose, and route-to-route extrapolations [1]. However, most regulators still hesitate to use PBK models to support regulatory decision-making. According to the results of an international survey, barriers to regulatory acceptance include a lack of modelling expertise in regulatory agencies to review submitted models, a lack of *in vivo* kinetic data to develop models, a lack of user-friendly platforms for reviewers to test the models, and differences in acceptance criteria between agencies and countries [2], [3]. Traditionally PBK models have been calibrated and evaluated with reference to *in vivo* kinetic data in test species. As the toxicity testing paradigm shifts to alternative testing approaches, there is also an increasing need to develop PBK models that rely (mostly or entirely) on model parameters quantified by using *in vitro* or *in silico* methods (so called “next generation” PBK models). Existing guidance documents on the development and application of PBK models have required the use of *in vivo* kinetic data collected from the test species for model parameterisation [1], [4], [5], and thus a new guidance for calibrating and evaluating PBK models developed without *in vivo* data was needed.

To address this gap, in 2017, the Organisation for Economic Cooperation and Development (OECD) initiated a project and formed an expert group to identify criteria to evaluate PBK models built using alternative approaches (such as *in vitro* data and/or *in silico* predictions) for chemicals that do not have available *in vivo* data for model parameterisation and validation (concordance assessment between model predictions and tissue/blood concentration data). The need for such a guidance document had been identified during a European Commission Joint Research Centre (JRC) EURL ECVAM workshop on “Physiologically-Based Kinetic modelling in risk assessment – reaching a whole new level in regulatory decision-making” held in 2016 [3], [6].

The OECD expert group, comprising more than 45 scientists and risk assessors, drafted a guidance document for characterising, validating, and reporting PBK models for regulatory purposes.

The OECD guidance document [7] includes a scientific workflow for characterising and validating PBK models, with emphasis on the use of *in vitro* and *in silico* data, and an assessment framework for evaluating models, with emphasis on identifying major uncertainties underlying model inputs and outputs. To help end-users submit or evaluate a PBK model submitted for regulatory purposes, the guidance document also includes a template for documenting a model, and a checklist for evaluating its quality. While the guidance provides contextual information on the scientific process of PBK model characterisation and validation, it is not intended to provide technical guidance on PBK model development or best practices for modellers. The guidance is broadly applicable to PBK models developed for humans, laboratory test species (e.g. rats, mice, dogs and rabbits), farm animals and species of ecological relevance (birds, fish, etc.).

In this commentary, we give an overview of the principles and criteria underpinning PBK models laid out in the OECD guidance document, with the aim of facilitating the dialogue between model developers and risk assessors.

## 2 Core of the PBK model guidance

Ideally, to facilitate and promote the acceptance of a PBK model that relies on *in vitro* or *in silico* data in regulatory risk assessment, a dialogue should be established between modellers and risk assessors on when and how to use PBK models developed for a specific purpose. The OECD guidance [7] aims to facilitate the dialogue between these two parties by offering a harmonised assessment framework, acceptance criteria, a workflow and tools for sharing the information related to the modelling process, including the strategy for model calibration and validation without *in vivo* kinetic data.

When *in vivo* data kinetic are not available for PBK model calibration, there are two key pre-requisites to parameterise a model:

1. Use a PBK modelling platform that contains/predicts values for all model parameters for a given chemical; or
2. Use customised *in vitro* and *in silico* methods to generate data of sufficient quality to parameterise a model.

**Fig. 1:**
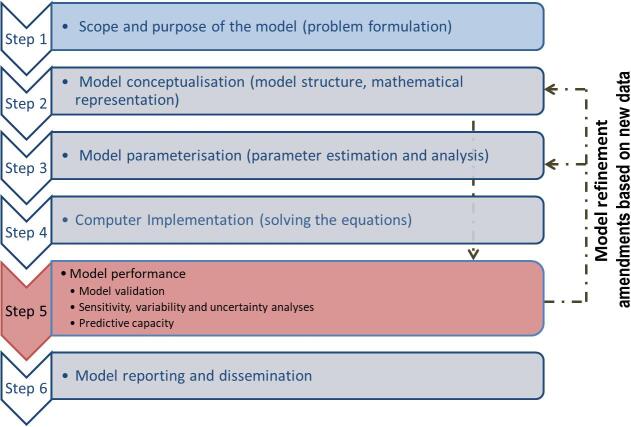
Schematic representation of the steps for PBK model development, validation, reporting and dissemination reported in the OECD guidance document. Figure taken from OECD [7].

The guidance provides a six-step workflow with emphasis on currently available tools and methods for model parameterisation (Fig. 1):

Step 1) Problem formulation: The regulatory purpose(s) of the model should be identified, along with the decision making context.

Step 2) Model conceptualisation (model structure and mathematical representation by equations): The structure of a PBK model should be informed by the problem formulation, knowledge of the underlying biokinetic mechanisms, and the availability of suitable data. The level of detail in model structure relates not only to the selection of compartments (e.g. whole body, target tissue or intracellular concentrations), but also to the chemical forms that are tracked within the model (i.e. parent chemical and/or metabolite(s)). The model should be only as complex as necessary to address the assessment in a fit-for-purpose manner. In some cases, a simple one-compartment model that describes the uptake and clearance of a chemical may be sufficient for screening of chemicals in risk assessment. In other cases, a PBK model that consists of two or more organ/tissue compartments is needed, for example, to include a target tissue when translating an *in vitro* dose measured using cells/enzymes from the target tissue (e.g. hepatocytes, thyroid peroxidase). The PBK model construction should also consider the exposure scenario/dosing strategy, as determined in the problem formulation step, taking into account inter-individual variability associated with some physiological parameters.

Step 3) Model parameterisation, estimation and analysis: PBK models are built using two sets of parameters: i) physiological and anatomical parameters, with representative reference parameters taken from the species under study (animal or human); and ii) physicochemical parameters, which are experimentally derived using *in vitro* methods or obtained using *in silico* approaches such as quantitative structure–activity relationship (QSAR) models. Key considerations for quantifying the most common absorption, distribution, metabolism, excretion (ADME) parameters are highlighted in the guidance document. Information on how these parameters are measured or predicted, and pointers for the modeller and assessor, are provided.

Step 4) Computer implementation: This includes the choice and description of the software used for execution of model code, as well as documentation of the model code.

Step 5) Model performance: In the absence of *in vivo* kinetic data, one approach for establishing confidence in the predictive ability of a PBK model is to analyse the predicted kinetics for chemical analogues, for which *in vivo* kinetic data are available. In effect, the predictive ability of the model for one or more analogues (“source chemicals” for which biokinetic data are available) is used to demonstrate the applicability of the model to the chemical of interest (“target chemical”) with similar ADME-relevant properties (but for which biokinetic data are lacking). Uncertainty and sensitivity analyses should also be carried out to evaluate model performance. The modeller may go back to step 2 and/or step 3 if the model performance is not considered adequate, and new information can be generated to improve the model performance (such as collecting new *in vitro* data, new biological knowledge).

Step 6) The final step is to report and disseminate the model and simulation results.

## 3 Assessment framework for PBK models

When *in vivo* data are not available for model validation, the following considerations can be followed to evaluate whether a model is appropriate for specific regulatory purposes:

### 3.1 Context and implementation

**Fig. 2:**
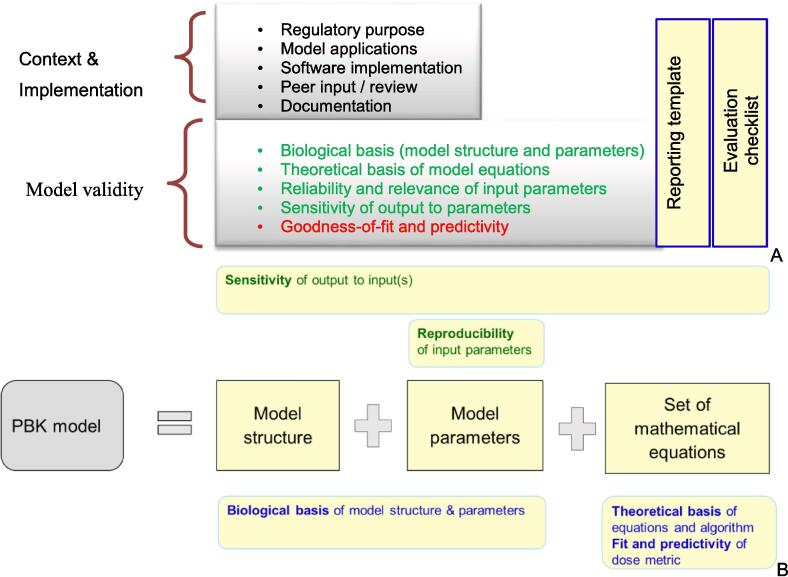
A. Characteristics of an Assessment Framework for PBK Models. Key information and supporting references are addressed in the model reporting template (by the model developer/proponent), while key questions to facilitate confidence assessment in the purpose-specific application of a model are outlined in the evaluation checklist (for use by the assessor/regulator). Considerations (under model validity) in green text do not require *in vivo* data; those in red require *in vivo* data. B. The scientific validity (reliability and relevance) of a PBK model. Reliability considerations in green (above), relevance consideration in blue (below). Figure taken from OECD [7]. (For interpretation of the references to colour in this figure legend, the reader is referred to the web version of this article.)

One set of considerations addresses the regulatory purpose and context of use, model applications, software implementation, peer input/review, and documentation (Fig. 2A).

To allow better use of the criteria provided in the guidance document, a reporting template and a checklist are provided. The reporting template follows the six-step workflow to capture the description of the model used simulations, allowing for the model evaluation to be more efficient and consistent. The checklist enables the end user (risk assessor) to evaluate the PBK model. Finally, a graphical template for ascribing relative confidence in a PBK model for a specific application is recommended. The confidence in a PBK model is considered high if: 1) its structure and parameters have reasonable biological basis (biological basis); 2) the model has been tested against biokinetic data in the species of interest and/or using analogues (model predictivity); and 3) the uncertainty of the predicted dose metric(s) has been established based on sensitivity analysis. The rationale for confidence determination should be included in accompanying text (examples are provided via a series of case studies).

### 3.2 Model validity

Another set of considerations are intrinsic to the model itself, including the biological basis of the model, theoretical basis of model equations, reliability and relevance of input parameters, sensitivity of output parameter(s), and finally goodness-of-fit and predictivity (Fig. 2B).

## 4 Proposed “alternative” approaches for validation

The document describes several approaches to access the quality of PBK models that cannot be evaluated by comparing predictions with *in vivo* data. These approaches include: 1) uncertainty and sensitivity analysis; 2) use of microscale systems; and 3) use of a read-across approach.

### 4.1 Uncertainty and sensitivity analysis

The quality (relevance and reliability) of an input parameter derived from cell and tissue-based *in vitro* methods should be assessed. To this end, the OECD Good In Vitro Method Practices (GIVIMP) guidance [8] highlights key elements of *in vitro* methods that are important when assessing the reliability of *in vitro* data. To foster confidence in *in vitro* alternatives to animal testing, the test methods and conditions under which data are generated must adhere to defined standards to ensure resulting data are reproducible.

Uncertainty and Sensitivity Analyses are two important techniques in the validation of any mathematical model, including a PBK model, and are typically carried out together. The aim of uncertainty analysis is to determine the overall uncertainty in model output, given the uncertainties in the model input parameters. The aim of sensitivity analysis is to quantify how much of the overall uncertainty in the model output can be attributed to each input parameter. Sensitivity analysis allows the uncertainty in model output to be ascribed to one or more input parameters within the model, thereby offering a means of ranking the parameters based on their relative contribution to the model output [9].

### 4.2 Microphysiological systems

Organ-on-Chip (OoC) models aim to recapitulate aspects of human physiology and pathology for use in drug discovery, efficacy and safety testing, and personalised medicine, with the goal to improve upon existing bioassays and provide insights into the mechanisms underlying the development and progression of diseases. In addition, OoCs are considered relevant to reduce the need for animal studies [10].

Although still in their infancy, it can be anticipated that OoC models, also known as microphysiological systems (MPS), will eventually provide an experimental basis for parameterising PBK models, especially in cases where *in vivo* data are lacking, and where there is a need to overcome drawbacks with current *in vitro* (static) systems. These systems could also be applied to validate PBK model predictions.

### 4.3 Read across approaches

In the absence of *in vivo* kinetic data, the use of analogues with available *in vivo* data provides a means of establishing confidence in the predictive ability of a PBK model. In effect, the predictive ability of a PBK model developed and validated for one or more analogues (“source chemicals”) can be used to predict time concentration dose metrics for a chemical of interest (“target chemical”) with similar ADME-relevant properties (but for which *in vivo* biokinetic data are lacking). The process for selecting suitable analogues, and choosing a suitable metric for judging similarity, is equivalent to the use of analogues for filling data gaps by read-across. A stepwise workflow is provided in the guidance document and also applied by Paini et al. [11]. Analogues can be identified based on their structural, physicochemical and/or ADME properties. The availability of tools for identifying analogues, and characterising their properties, is described in several articles [12], [13].

## 5 Case studies

The OECD PBK model guidance document [7] also provides several case studies to demonstrate the applicability of the principles and tools outlined in the document. Thirteen case studies provide illustrative examples covering several applications in human and environmental risk assessment. The case studies cover both traditional and newer applications of PBK models, including:

• read across to model analogues applying the workflow described in the document;
• environmental and human (bio)monitoring, internal species sensitivity distributions;
• interspecies differences extrapolation;
• inter individual differences extrapolation;
• acute to chronic, high to low dose, and short to long term (animal vs human, occupational vs population) extrapolations;
• route to route extrapolation;
• deriving a Point of Departure for risk assessment using quantitative *in vitro* to *in vivo* extrapolation (QIVIVE) from estimated free *in vitro* concentrations;
• next generation risk assessment of dermally applied consumer products.

## 6 Conclusions

The OECD document [7] builds on previously developed guidance by multiple agencies and organisations including the US EPA [4], WHO [1], EFSA [14], CEN [15], EMA [5], US FDA [16], and Japanese PMDA [17]. However, the OECD guidance is the first to focus on characterising, validating and reporting of PBK models based exclusively on *in vitro* and *in silico* data for ADME parameters.

The guidance is applicable to PBK models for chemicals used in a range of products, except for medical devices and products where guidance is already established. In principle, the guidance is applicable to chemicals in the nanoform (nanomaterials; NMs), biologicals, macromolecules, and metals, but would need to be extended to capture the additional features relating to the kinetics of these compounds, and the status of non-animal methods to parameterise the models. An extension of traditional PBK modelling to cellular and subcellular compartments is in principle covered in the scope of this guidance, is the linkage to toxicodynamics measured *in vitro*. However, the stability of the test chemicals *in vitro* could be influenced by the experimental set-up (e.g. migration to plastic, binding with proteins and lipids and evaporation) and this will affect the cellular responses. Mimicking the actual chemical kinetics in cells in the target tissues under real-world exposure scenarios should therefore be taken into account [18], [19], [20], [21], [22].

The document is expected to help in bridging the model developer and user (in particular, risk assessors) communities by providing a common assessment framework with consistent terminology, thereby addressing barriers in communication. In addition, with the increasing advances in *in vitro* and *in silico* methodologies, the guidance document can be updated in the future to cover new aspects (e.g. nanomaterials, OoC models). By providing a harmonised and structured framework for modellers and risk assessors to communicate the characteristics of PBK models, it is expected that the guidance document will encourage the acceptance and uptake of PBK models in the risk assessment process.

## 7 Disclaimer

The opinions expressed and arguments employed herein are those of the authors and do not necessarily reflect the official views of the European Commission, the OECD or of the governments of OECD Member Countries.

## Declaration of Competing Interest

The authors declare that they have no known competing financial interests or personal relationships that could have appeared to influence the work reported in this paper.
